# Light Weight Deep Learning Algorithm for Voice Call Quality of Services (Qos) in Cellular Communication

**DOI:** 10.1155/2022/6084044

**Published:** 2022-08-30

**Authors:** Mritha Ramalingam, S. J. Sultanuddin, N. Nithya, T. F. Michael Raj, T. Rajesh Kumar, S. J. Suji Prasad, Essam A. Al-Ammar, M. H. Siddique, Sridhar Udayakumar

**Affiliations:** ^1^Faculty of Computing, College of Computing and Applied Sciences, Universiti Malaysia Pahang Pekan, Pahang 26600, Malaysia; ^2^Department of Computer Applications, MEASI Institute of Information Technology, Chennai 600014, Tamil Nadu, India; ^3^Department of Electronics and Communications Engineering, Panimalar Engineering College, Chennai 600123, Tamil Nadu, India; ^4^School of Computing Science and Engineering, Galgotias University, Greater Noida 201310, Uttar Pradesh, India; ^5^Department of Computer Science and Engineering, Saveetha School of Engineering, Saveetha Institute of Medical and Technical Sciences, Chennai 602105, Tamil Nadu, India; ^6^Department of Electronics and Instrumentation Engineering, Kongu Engineering College, Erode 638060, Tamil Nadu, India; ^7^Department of Electrical Engineering, College of Engineering, King Saud University, P.O. Box 800, Riyadh 11421, Saudi Arabia; ^8^Intelligent Construction Automation Centre, Kyungpook National University, Daegu, Republic of Korea; ^9^Department of IT, Mettu University, Mettu, Ethiopia

## Abstract

In this paper, a deep learning algorithm was proposed to ensure the voice call quality of the cellular communication networks. This proposed model was consecutively monitoring the voice data packets and ensuring the proper message between the transmitter and receiver. The phone sends its unique identification code to the station. The telephone and station maintain a constant radio connection and exchange packets from time to time. The phone can communicate with the station via analog protocol (NMT-450) or digital (DAMPS, GSM). Cellular networks may have base stations of different standards, which allow you to improve network performance and improve its coverage. Cellular networks are different operators connected to each other, as well as a fixed telephone network that allows subscribers of one operator to another to make calls from mobile phones to landlines and from landlines to mobiles. The simulation is conducted in Matlab against different performance metrics, that is, related to the quality of service metric. The results of the simulation show that the proposed method has a higher QoS rate than the existing method over an average of 97.35%.

## 1. Introduction

The first cellular networks were developed using analog first generation (1G) standards. The most common are NMT and AMPS. Usually, next to the standard name, they write the frequency in megahertz, followed by the frequency range for basic station communication with mobile phones. For example, the basic stations on NMT-450 networks communicate with cell phones on the 450 MHz bandwidth. Working simultaneously on analog standards to ensure that multiple mobile phones in a cell, as well as the base stations of different cells, used multiple frequency multiplexing (FDMA, frequency segment multiple access, simultaneous access with frequency segment), which would work in one of the conditions of scarcity of free frequencies [1]. The cellular range was a maximum of 10–20 phones and large cell sizes only. This is acceptable only because mobile communications are relatively limited. Also, analog standards provide no protection against interference, and sometimes the conversation can be heard using a simple radio receiver. In second-generation networks (2G, second generation), data are transmitted between base stations and mobile phones in digital format [2]. This enables the use of time division multiplexing (TDMA, time division multiple access, simultaneous access with time division) in DAMPS standards and multiple from one base station. Modified GSM to run phones simultaneously-each band channel is split. It is called timeslots, that is, the time intervals, at which the channel is occupied by a telephone [3]. Therefore, a base station can serve up to several hundred phones simultaneously. The power of transmitters has been reduced in second-generation mobile phones as the transmission loss of digital sound is very low [4].

CDMA (code segment multi-access) makes extensive use of sophisticated methods of separating radio air between different mobile phones. Also, no matter how many different phones are in one cell, and no matter how many base stations are in the neighborhood, each mobile phone uses the CDMA 2000 1x standard of 1.25 MHz to receive and transmit the entire frequency band (channel) of relatively large width [5, 6]. To distinguish signals from different telephones and base stations, each transmitter has its own code, which is transmitted over the entire channel width. The most popular cellular communication standard is the second-generation GSM-Global System for Mobile Communications (Global System for Mobile Communications) [7]. These quality mobile phones are now used by more than 1 billion people worldwide. But the main effect of the switch to the digital form of the signal is the ability to use mobile phones to transmit not only voice (audio) but also other types of information [8]. The first service that made it possible to exchange text messages between mobile phones was called service. With the help of SMS technology, you can send not only text messages but also simple pictures and sounds, as well as express your emotions using special images called emoticons (from smile-smile). It uses EMS and Nokia smart messaging technologies [9–11].

Later, with the advancement of mobile phones and the development of computerization, technologies for GSM networks such as computer data transfer and Internet access were introduced (cm Internet) [12]. The first such technology was CST (Circuit Switched Data, Direct Connection via Data Transmission), which was assigned to the phone. These models are used to transfer data at a speed of 9.6 kilobits per second—the same time allotment is made when making phone calls. In this case, the phone cannot be used for its intended purpose [13]. To increase the transfer speed, HSCST (high-speed CST, high-speed CST) technology was developed—the phone receives multiple time intervals simultaneously, and a special algorithm is used to correct errors depending on the quality of the connection [14]. With this technology, there may not be enough time intervals for all mobile phones in a cell, so this is not common.

The most common data transfer technology is GPRS (General Pocket Radio Service). GPRS allows multiple mobile phones to use dedicated time intervals simultaneously, using different algorithms for different quality of communication with BS and different workloads of BS [15]. Each phone uses different time intervals, releasing them when not needed or requesting new ones. Using patch splitting, the time slots between phones are divided into computer networks. The number of time intervals the phone can use is determined by the hardware and depends on the GPRS class of the mobile phone [13, 16]. Transfer speeds are asymmetrical with the 8th and 10th GPRS classes. If a class phone uses 4-time intervals to get information, there are only 1-2 for transfer. The GPRS speed is 107 kilobits per second with a theoretical maximum connection speed of 21.4 kilobits per second and 5 allotted time intervals. But in reality, the average speed of GPRS is 56 kilobits per second. When using GPRS technology, mobile phones are assigned IP addresses over the Internet, which in most cases are not unique. Edge technology is the next step in the evolution of GPRS technology (improved data rates for GSM evolution, faster data transfer for GSM development) [17–19]. In this technology, new information encoding schemes have been used compared to GPRS, and the error handling algorithm has been changed (incorrectly sent packets are not sent back, only information for their recovery is sent) [20]. As a result, the maximum transfer rate reaches 384 kilobits per second. GPRS technology is sometimes referred to as Generation 2.5–2.5 G mobile communication technology, and EDGE technology as 2.75 G technology. For CDMA2000 networks, 1xRTT technology has been developed that allows speeds of up to 144 kilobits per second [21].

Initially, these technologies were used on mobile phones to access the Internet using personal computers, and only later, did further development of mobile phones provide direct Internet access from mobile phones. To obtain information on a mobile phone, WAP technology (Wireless Application Protocol, a protocol for wireless applications), which has relatively small requirements for technical specifications [22], pages created with special language WML (Wireless Markup Language) is unique to mobile phones: small screen size, keyboard control only, low-speed data transfers, page load delays, and more. Also, due to the low performance of the processor and the small amount of memory of the mobile phone, the mobile browser does not process pages directly in this language for maximum functionality but compiles them into a special byte code, that is, executed by the mobile phone with the help of an intermediate server (known as the WAP gateway) [23]. This is why the work of an intermediate server is highly valued by mobile operators.

However, with the advancement of mobile phones, things changed quickly. First, the need for an intermediate server has disappeared—now the browsers of modern mobile phones do their work independently. Second, the specialized language WML is replaced by the XHTML standard, which differs from the language commonly used on the Internet [24]. It is only by following certain rules of the HTML language, that is, the XML specification. Third, modern mobile phones have a screen size large enough to display normal web pages for computers. Fourth, with the development of the modern Internet, the code of HTML pages has now begun to be simplified and configured primarily due to machine writing [25]. Due to these changes, many modern phones are capable of handling HTML themselves.

The main contribution of the paper includes:

- The authors devised a deep learning algorithm was proposed to ensure the voice call quality of the cellular communication networks.
- The deep learning model consecutively monitors the voice data packets and ensures the proper message between the transmitter and receiver.

## 2. Literature Review

Carvalho et al. [1] discussed the honeycomb area that joins together to form a network. On an ideal (even without a building) surface, the coverage area of a BS is a circle, so the network formed by them looks like a honeycomb with hexagonal cells (honeycomb). The network has intermittent transceivers that allow you to determine the current location of mobile subscribers, switch devices, and ensure communication continuity as a subscriber moves from one coverage area to another. Logeshwaran et al. [2] talked about cellular networks. Different operators are connected to each other as well as to a fixed telephone network. It allows subscribers of one operator to make subscriptions to another operator from mobile phones to landlines and from landlines to mobiles. Operators from different countries can complete roaming contracts. Papazoglou et al. [4] talked about how, in a cellular system, a powerful stationary base station above the city center can be replaced by a number of identical low-power stations installed close to the ground.

Cells that use the same radio channel group can avoid interruptions if they are spaced apart. In this case, frequency reuse is observed. Cheng et al. [5] expressed frequency replication is the assignment of the same frequencies (channels) to multiple cells if these cells are separated by significant distances. Frequency reuse is facilitated by reducing the coverage area of each cell. The base station of each cell receives a group of different working frequencies from neighboring cells, and the base station antennas are selected to cover the desired coverage area within its cell.

According to Hsu et al. [6], the coverage area is limited to the boundaries of a cell, and different cells can use the same set of operating frequencies without mutual influences if they are sufficiently separated from each other. Deoskar [8] discussed that based on these data transfer technologies, additional services are available for mobile phones, for example, MMS (Multimedia Messaging System). Using a mobile phone, you can now easily create a message containing text, images, audio, video, or more. Technically, when an MMS message is sent, a dedicated data transfer protocol is used over a regular Internet connection such as GPRS. MMS messages can be sent from a mobile phone not only to other mobile phones but also to e-mail addresses. An E-mail will receive all the files that makeup MMS. Each message can be sent to multiple addresses simultaneously.

Hussien and Sadi [9] explained that if the addressee is the number of another mobile phone that supports MMS, he or she will download the contents of the message automatically or directly using a special protocol upon special request. If the receiving mobile phone does not support MMS, it receives an SMS message with a link on the Internet; by clicking on it, you can view MMS content over the Internet from your mobile phone or personal computer. However, most modern mobile phones are equipped with programs such as e-mail clients, and as they upgrade, MMS becomes redundant and is replaced by other services. Braden et al. [10] discussed how the MDA maintains such intercom sessions with the base station whenever the cell phone is on. Initially, this happens after the MTA's power is activated, and then the telephone communicates with its operator's nearest telecommunications station, stabilizes its position on the ground, and transmits its data. It is registered on the network. Based on this record, during subsequent negotiations, this subscriber will be charged for connections, communication services, call charges, and roaming. In addition to the time intervals of the communication session when the power is on, the MTA communicates with the nearest base station once an hour, stabilizing its position and, if necessary, at the zonal charge of another neighboring base station.

Ezaki et al. [12] discussed how the duration and frequency of service contact sessions vary across MTAs, ranging from 10 to 35 times per day. In this case, the duration of the time intervals will vary in the range of 2–25 milliseconds. In many modern MDAs, the functions of the various types of services that inform the owner will be activated automatically, for example, about the weather forecast or messages, so the time intervals for such a phone will be frequent and long. In this case, without special equipment, it is not possible to determine what kind of signals your mobile phone sends to the base station.

From the existing models, it is found that most of the communications are not analyzed at the micro-level and even the micro-level (optimal call admission control (OCAC), dynamic channel assignment algorithm (DCAA), integrated structured cabling system (ISCS), and fuzzy probabilistic based semi-Markov model (FPSMM)) solutions provide a computational burden to the entire system. This leads to increased computational cost and communication cost in estimating the solution related to voice call QoS.

## 3. Proposed Model

The data transfer rates on second-generation networks are not sufficient to carry out many new functions of mobile communications, especially high-quality video in real-time (videophone), modern optical computer games on the Internet, and others. New standards and protocols have been developed to ensure the required speed:

- The UMTS standard (Universal Mobile Telecommunications System, Global System for Mobile Communications) is based on W-CDMA technology (wideband code multiple access, broadband CDMA), somewhat compatible with GSM. The speed of data reception and transmission reaches 1920 kilobits per second.
- 1xEV technology (evolution, development) for CDMA2000 networks. Data reception speeds reach 3.1 megabits per second, and transfer speeds reach 1.8 megabits per second.
- Technologies like TD-SCMA, HSDPA, and HSUPA. It allows you to reach even higher speeds. Until 2006, W-CDMA technologies mostly provided HSDPA support. TD-SCMA is in development.

In this way, modern technologies such as mobile communication are not mobile phone technologies like global communication technologies. Microcells are commonly used in densely populated areas. Due to their small range, microcells are less susceptible to transfer degradation effects such as reflection and signal delays. A macrocell integrates with a group of microcells. The microcell serves slow-moving mobile devices and the macrocell serves fast-moving devices. The mobile device can determine the speed of its movement. This makes it possible to reduce the number of hops from one cell to another and adjust the location data.

When the distance between the mobile device and the base station of the microcell is small, the transition mechanism from one cell to another can be changed. When planning systems using hexagonal cells, the base station transmitters are located at the center of the cell, at the edge of the cell, or at the top of the cell. Cells with a transmitter at the center typically use omnidirectional antennas, and cells with transmitters at the edge or apex typically use sector directional antennas. Consider a system with a fixed number of full-duplex channels in an area. Each service area is split up into groups called “clusters.” Each cluster gets a group of channels that are spread out among the cells of the cluster in different ways.

(1) $$\begin{array}{c} N=A*D, \end{array}$$

where *N* = the number of complete dual cellular channels available in the cluster; *A* = the number of channels in the cell; and *D* = the number of cells in a cluster.

If the cluster is duplicated within the specified service area, then the total number of full-duplex channels is :

(2) $$\begin{array}{c} M=kA*D, \end{array}$$

where *M* = total number of channels in a given region; *A* = the number of channels in the cell; *D* = the number of cells in a cluster; and *k* = the number of clusters in a given zone.

From the exposures, we can see that the total number of channels in the cellular telephone system is directly proportional to the number of back clusters in a given service area. If the cluster size decreases but the cell size does not change, more clusters will be needed to cover a given service area, and the total number of channels in the system will increase. Due to the absence of neighboring cells in a small service area (for example, within a city), the number of subscribers, who can use the same frequencies (channels) simultaneously depends on the total number of cells in the area. The average number of such subscribers is four, but in densely populated areas, the number is much higher.

With the planned increase in cellular transport, the increased demand for service is met by reducing the size of the cell, which divides it into several cells, each with its own base station. If the cells are not very small, efficient cell division allows the system to handle more calls. If the cell diameter is less than 460 m, the base stations of neighboring cells will affect each other. The criterion for determining the relationship between frequency reuse and cluster size is the cellular structure as the subscriber density increases.

**Figure 1:**
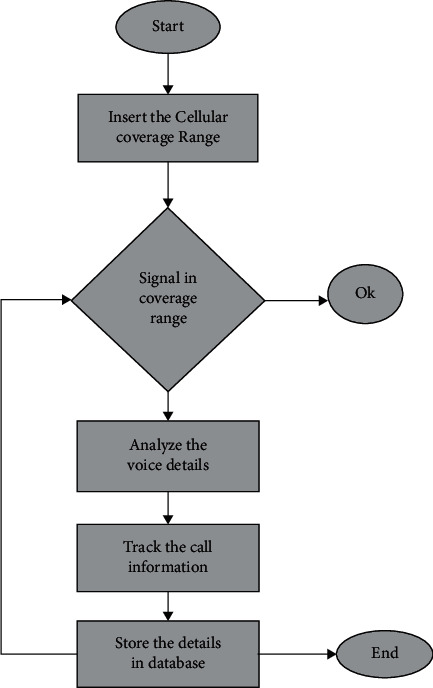
Proposed model flow diagram.

If the number of cells in a cluster is small, the chances of interactions between channels are high. Because cells are hexagonal, each cell will always have six equidistant neighbors, and the angle between the lines connecting the center of any cell with the centers of neighboring cells is 60. Therefore, the number of possible cluster sizes and cell configurations is small, as shown in Figure 1. To connect cells without gaps (mosaic), the hexagon's geometric measurements must be such that the number of cells in the cluster meets the following condition :

(3) $$\begin{array}{c} S=z^{2}+zx+x^{2}, \end{array}$$

where *S* = the number of cells in the *S*-cluster; *z* and *x* = non-negative integers (Algorithm1).

The first step is to set up the cellular channel. Examine the information about the signal in line with the standards for the highest possible number of cellular channels in the input signal density. A count is kept for each and every channel. It is necessary to perform an update on both the z-value and the x-value. If the channel index is one, low signal values are more than the maximum channel index, and you adjust the value of the channel input signal index such that it is different. In the event that the signal values are greater than the maximum channel index, then the random channel index of the signal input is selected. Make the necessary adjustments to the values of *z* and *x* in accordance with the requirements of the new channel. Take a reading of the signal strength, then evaluate it in relation to the channel index values. It is necessary to offer the findings of the classification of the many facets of the voice.

**Figure 2:**
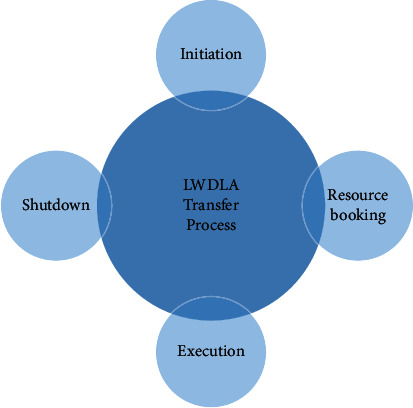
Proposed model transfer process.

The transfer process from one cell to another, i.e., when the mobile device moves from the base station 1 to base station 2, has four main steps shown in Figure 2:

- Initiation-detects the need of the mobile device or network and initiates the necessary network procedures;
- Resource booking: with the help of appropriate network procedures, network resources are allocated, which are necessary to change the service (voice channel and control channel);
- Execution: direct transfer of control from one base station to another;
- Shutdown: unwanted network resources are released and available to other mobile devices.

Mobile communication, also known as cell phones, is carried out by radio waves without the use of wires, as in a regular telephone system:

(4) $$\begin{array}{c} Q=\sum_{e=1}^{r}\sum_{y=1}^{t}u_{y}^{\left(e\right)}-k_{e}^{2}\;, \end{array}$$

where the above equation (1), *Q*–the cellular utilization point, *R*–the range cellular cluster, *t*–the quantity of signal, *u*_*e*_^(*e*)^—*y*^th^ case of *e*^th^ cellular cluster, *K*_*e*_–the central cellular of *e*^th^ cluster.

The automatic telephone transmission first monitors the car's movement in the station zone. During a call, if the machine skips one zone and enters the next, the call will automatically be transferred to the base station operating in that zone. When making a mobile phone call, the caller connects to an automated cellular telephone exchange that detects the mobile phone, requests a free radio channel from the circuit controller, and communicates with the desired number via the base station. Then the cell phone calls, and when the driver picks up the phone, the circuit is complete.

## 4. Results and Discussion

The proposed lightweight deep learning algorithm (LWDLA) was compared with the existing optimal call admission control (OCAC), dynamic channel assignment algorithm (DCAA), integrated structured cabling system (ISCS), and fuzzy probabilistic based semi-Markov model (FPSMM). The entire simulation is conducted in a Matlab environment that runs on a high-end computing system with 8 GB of RAM and an i5 computing processor.

### 4.1. Channel Location Management

The moving vehicle's location is found by the automatic telephone transmission, while the call is sent to the communication channel by the circuit controller.  Table 1 shows the evolution of channel location management.

In the cutoff region of the channel location area, all the existing models achieved 63.05%, 71.32%, 63.36%, and 87.24% of channel allocations. But the proposed model achieved 92.08% of the channel allocation. This is possible because the proposed model already predicted the upcoming signal location. Hence, this achieves a high allocation rate.

### 4.2. Cellular Area Management

When the vehicle leaves the area of the remote base station, the driver cannot use cellular communications. If the call is made on the way to the boundary of the zone, the signal will weaken and eventually disappear completely.  Table 2 shows the evolution of cellular area management.Where the cutoff region of the cellular location of all the existing models was achieved at 63.35%, 73.62%, 59.96%, and 84.50% of channel location management. But the proposed model achieved 89.99% of location management. This is possible because the proposed model already predicted the upcoming area pointer. Hence, this achieves the exact location of the signal.

### 4.3. Station to Station Management

Where the cutoff region of the base station management for all the existing models was achieved at 64.90%, 65.48%, 58.43%, and 79.86% of base station management. But the proposed model achieved 93.88% of base station management. This is possible because the proposed model was already predicted for the base station. It is useful for the users to periodically modify the base stations while traveling. Hence, this achieves high base station management.  Table 3 shows the evolution of station to station management.

### 4.4. Data Transfer Rate

In most communication networks, the transmitter transmits the higher data rate messages through the air medium. This was always at a higher level because, based on the modulation and filter quality, this may be affected. So the data rate from the transmitter was always huge. From the receiver end, the same data rate was received, and then the demodulation process was performed with the help of different filters.  Table 4 shows the evolution of data transfer rate.

Where the cutoff region of the data transfer rate for all the existing models was achieved, 56.23%, 68.98%, 57.61%, and 79.87% of transfer communication between the transmitter and receiver. But the proposed model achieved 92.36% of the channel allocation. This is possible because the proposed model already found the shortest path between two endpoints. Hence, this achieves a high data transfer rate.

### 4.5. Communication Speed

In general, the communication mediums delay the process. The delay from the transmitter is called the transmission delay, and the delay from the receiving medium is called the receiver delay. So the speed of the transmission and receiving medium are always high for cellular communication.  Table 5 shows the evolution of communication speed.

In the cutoff region of the communication speed, all the existing models achieved 63.09%, 64.79%, 54.57%, and 75.54% of channel allocations. But the proposed model achieved 96.70% of communication speed. This is possible because the proposed model already predicted the upcoming destination. Hence, this achieves a high speed.

### 4.6. Quality of Services

In general, the quality of service in cellular communication is very essential because this is the parameter used to evaluate the service range of the cellular base station. These cellular communication instruments are performed well to increase the quality of service of the network.  Table 6 shows the evolution of quality of services.Where the cutoff region of the quality of service for all the existing models was achieved, 65.34%, 43.60%, 57.94%, and 80.35% of channel allocations. But the proposed model achieved 88.68% of QoS. This is possible because the proposed model already predicted the upcoming channel operation. Hence, this achieves the quality of service.

## 5. Conclusion

A modern MTA can be automatically switched to Dictaphone mode by a signal or a given program without its owner's approval. It is not true that every MDA records the owner's speech and voice and then sends the information, but such an opportunity is technically provided in every modern MDA. The proposed LWDLA was compared with the existing OCAC, DCAA, ISCS, and FPSMM. If an action takes place during a show in a theater, it is almost obvious that the gun will explode by the end of the show. So, in this case, MTA has the ability to record and transfer information, and this factor should be taken into account when using your mobile phone. The MTA receives information from the station closest to the cell. In it, information spreads in the air. The MTA interacts with the station at bursts of digital pulse signals called “time intervals.” The duration of a service contact session can range from fractions of a second to several seconds. In the future, the solutions can be improved by increasing the rate of QoS of voice calls or HD voice calling using artificial intelligence approaches.

## Figures and Tables

**Algorithm 1:**
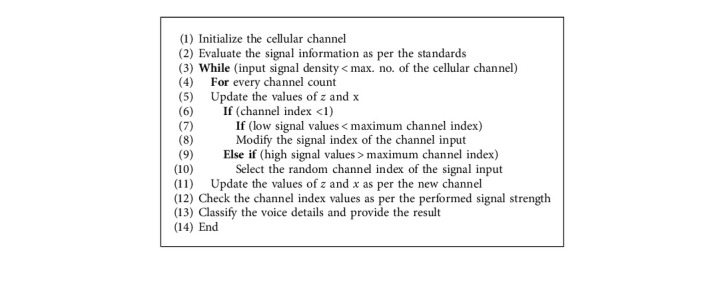
Lightweight deep learning algorithm (LWDLA).

**Table 1:** evolution of channel location management.

No of channels	OCAC	DCAA	ISCS	FPSMM	LWDLA
1000	60.24	68.33	60.58	83.63	89.95
2000	60.57	69.83	61.17	85.50	90.99
3000	61.91	70.94	62.15	86.33	91.12
4000	63.05	71.32	63.36	87.24	92.08
5000	64.10	72.33	64.50	88.16	91.65
6000	64.81	73.26	65.61	89.49	92.89
7000	66.11	74.26	66.31	90.36	93.00

**Table 2:** evolution of cellular area management.

No of channels	OCAC	DCAA	ISCS	FPSMM	LWDLA
1000	62.54	70.63	57.18	80.89	87.86
2000	62.87	72.13	57.77	82.76	88.87
3000	64.21	73.24	58.75	83.59	89.03
4000	65.35	73.62	59.96	84.50	89.99
5000	66.40	74.63	61.10	85.42	89.56
6000	67.11	75.56	62.21	86.75	90.76
7000	68.41	76.56	62.91	87.62	90.91

**Table 3:** evolution of station to station management.

No of channels	OCAC	DCAA	ISCS	FPSMM	LWDLA
1000	61.50	60.59	53.02	75.19	90.69
2000	63.13	62.33	54.60	76.61	91.98
3000	63.61	64.67	56.80	77.87	92.99
4000	64.90	65.48	58.43	79.86	93.88
5000	67.01	67.77	59.57	82.33	94.25
6000	68.50	69.70	61.77	83.77	95.89
7000	70.31	71.43	62.92	85.49	96.26

**Table 4:** evolution of data transfer rate.

No of channels	OCAC	DCAA	ISCS	FPSMM	LWDLA
1000	51.61	64.69	53.18	76.20	90.69
2000	53.10	66.66	55.60	78.40	90.68
3000	53.90	67.79	56.01	79.20	91.88
4000	56.23	68.98	57.61	79.87	92.36
5000	57.24	69.37	59.93	81.30	93.79
6000	57.88	70.89	61.18	82.39	94.95
7000	58.54	71.13	63.91	82.87	95.72

**Table 5:** evolution of communication speed.

No of channels	OCAC	DCAA	ISCS	FPSMM	LWDLA
1000	60.12	61.06	50.67	72.00	93.85
2000	60.01	61.08	50.50	71.73	93.35
3000	59.99	61.96	51.23	72.03	93.47
4000	63.09	64.79	54.57	75.54	96.70
5000	64.29	66.11	55.30	76.86	97.08
6000	64.90	66.94	56.19	77.40	97.65
7000	65.31	67.34	56.27	77.70	97.35

**Table 6:** evolution of quality of services.

No of channels	OCAC	DCAA	ISCS	FPSMM	LWDLA
1000	60.72	39.31	53.51	76.68	87.01
2000	62.21	41.28	55.93	78.88	87.00
3000	63.01	42.41	56.34	79.68	88.20
4000	65.34	43.60	57.94	80.35	88.68
5000	66.35	43.99	60.26	81.78	90.11
6000	66.99	45.51	61.51	82.87	91.27
7000	67.65	45.75	64.24	83.35	92.04

## Data Availability

The data used to support the findings of this study are included in the article. Further data or information is available from the corresponding author upon request.
